# A Novel Viewpoint on the Anticipatory Postural Adjustments During Gait Initiation

**DOI:** 10.3389/fnhum.2021.709780

**Published:** 2021-10-11

**Authors:** Veronica Farinelli, Francesco Bolzoni, Silvia Maria Marchese, Roberto Esposti, Paolo Cavallari

**Affiliations:** ^1^Human Physiology Section of the Department of Pathophysiology and Transplantation, Università degli Studi di Milano, Milan, Italy; ^2^Department of Biomedical Sciences, Humanitas University, Pieve Emanuele, Italy

**Keywords:** APAs, trunk muscles, prime movers, ankle muscles, postural control, human

## Abstract

Anticipatory postural adjustments (APAs) are the coordinated muscular activities that precede the voluntary movements to counteract the associated postural perturbations. Many studies about gait initiation call APAs those activities that precede the heel-off of the leading foot, thus taking heel-off as the onset of voluntary movement. In particular, leg muscles drive the center of pressure (CoP) both laterally, to shift the body weight over the trailing foot and backward, to create a disequilibrium torque pushing forward the center of mass (CoM). However, since subjects want to propel their body rather than lift their foot, the onset of gait should be the CoM displacement, which starts with the backward CoP shift. If so, the leg muscles driving such a shift are the prime movers. Moreover, since the disequilibrium torque is mechanically equivalent to a forward force acting at the pelvis level, APAs should be required to link the body segments to the pelvis: distributing such concentrated force throughout the body would make all segments move homogeneously. In the aim of testing this hypothesis, we analyzed gait initiation in 15 right-footed healthy subjects, searching for activities in trunk muscles that precede the onset of the backward CoP shift. Subjects stood on a force plate for about 10 s and then started walking at their natural speed. A minimum of 10 trials were collected. A force plate measured the CoP position while wireless probes recorded the electromyographic activities. Recordings ascertained that at gait onset APAs develop in trunk muscles. On the right side, Rectus Abdominis and Obliquus Abdominis were activated in 11 and 13 subjects, respectively, starting on average 33 and 54 ms before the CoP shift; Erector Spinae (ES) at L2 and T3 levels was instead inhibited (9 and 7 subjects, 104 and 120 ms). On the contralateral side, the same muscles showed excitatory APAs (abdominals in 11 and 12 subjects, 27 and 82 ms; ES in 10 and 7 subjects, 75 and 32 ms). The results of this study provide a novel framework for distinguishing postural from voluntary actions, which may be relevant for the diagnosis and rehabilitation of gait disorders.

## Introduction

Walking is one of the most common and natural motor actions performed in daily life. However, this naturalness requires a prolonged learning period in which the automatic stepping activity must be coupled with a postural control under development (Forssberg, 1999). The intrinsic complexity of this motor behavior is apparent when starting to walk (i.e., gait initiation), where the control of focal movement should be strictly coupled to a feedforward postural control. This has been described both in healthy children (Assaiante et al., 2000) and in patients suffering from different neurological conditions (Delval et al., 2014; Delafontaine et al., 2019; Farinelli et al., 2020; Palmisano et al., 2020). The “complexity of simplicity” becomes evident even to the walker when the physiological organization of the neural network underlying gait initiation is impaired, causing a dramatic deterioration of the self-confidence of the patient and increasing the risk of fall (Walton et al., 2015).

Before initiating a movement, throughout a top-down control, our brain shapes specific feedforward motor programs dispatched to the prime movers and to the postural muscles. The ensuing postural activities that precede the onset of the voluntary movement are called anticipatory postural adjustments (APAs). According to the literature (for a review, refer to Massion, 1992; Bouisset and Do, 2008; Cavallari et al., 2016), APAs are defined as muscular activities intended to prevent the mechanical perturbations caused by the focal movement, by building up fixation chains that link the body segments to the available support points. If not properly counterbalanced, these perturbations would affect, or even impede, the correct execution of the voluntary movement itself.

Gait initiation seemingly introduces an exception to this well-accepted definition of APAs. In fact, in this specific case, the literature calls APAs those muscular activities that produce a specific well-tailored perturbation of the equilibrium, i.e., the forward displacement of the center of mass (CoM) of a body, which is necessary to make the first step. This dichotomy in the definition of APA opens a question: are APAs involved in preserving the equilibrium or in disturbing it, or are they capable of both? What it may appear as a mere semantic issue becomes a crucial matter when studying gait initiation; in fact, the nervous system organizes the postural and voluntary component of the movements in different ways, and different involvements of these components may imply evident clinical repercussions (Takakusaki, 2017; de Lima-Pardini et al., 2020). For example, according to the hypotheses summarized in the review of Takakusaki, both the motor program for voluntary skilled movements and the program for the associated postural actions are processed with the contribution of the supplementary motor area (SMA) and premotor cortices. Then, the voluntary command is forwarded to the primary motor cortex (M1) and reaches the focal muscles through the lateral corticospinal tract, while the APA command follows the corticoreticular projections and reaches the postural muscles through the reticulospinal tract. This highlights how much the correct categorization of many muscular activities within a given action, distinguishing what is volitional and what regards postural control, would be relevant for a correct diagnosis of motor disturbances. Therefore, is it true that APAs play a different role in gait initiation than in other motor actions?

Approaching such “APA dualism” requires an insight into how the voluntary movement integrates with the postural actions. Prior to the onset of a voluntary movement, it is possible to identify changes in the activity of muscles directly responsible for the action (i.e., the prime movers) and that of muscles acting on the body support (i.e., postural muscles). The distinction between prime movers and postural muscles is apparent when considering upper limb movements in which postural muscles have a clear role in preserving the whole-body equilibrium (Bouisset and Zattara, 1981; Aruin and Latash, 1995). This distinction becomes less evident when a voluntary movement requires a change in the whole-body position, such as in gait initiation. In this case, the muscles acting on the body support surface produce mechanical actions that are fundamental for initiating the intended movement. It is also worth noting that while APAs preserving body equilibrium usually precede the voluntary movement by no more than about 200 ms, when initiating gait, the muscles acting on the support base are recruited much more in advance. Most of the literature agrees in identifying three phases in gait initiation: (i) the imbalance phase, which starts with the shift of the CoP backward and toward the future swing foot and ends with the heel-off; (ii) the unloading phase, from heel-off to toe-off of the swing foot, in which CoP shifts toward the future stance foot; and (iii) the swing phase, from toe-off to heel-strike, in which CoP moves forward (Crenna et al., 2006). Traditionally, the “APA” window coincides with the imbalance phase, lasting from the very first CoP shift to the onset of heel-off, thus covering a time period of around 500 ms for gait initiation at natural speed, down to 300 ms at maximal speed (Crenna and Frigo, 1991; Assaiante et al., 2000; for a review, refer to Yiou et al., 2017). This CoP movement is driven by the activities of ankle joint muscles: an inhibition of both Soleus (Sol) muscles, which are tonically active when standing, followed by the excitation of Tibialis Anterior (TA) muscles (about 100 ms later, for one of the first description, refer to Crenna and Frigo, 1991). Aiming at distinguishing these early activities, which alter the body equilibrium to ensure the adequate mechanical conditions for the planned action, from the classical APAs that preserve body equilibrium against the mechanical perturbation, Klous et al. (2012) called them early postural adjustments (EPAs) and also reported the different behavior of EPAs vs. APAs.

To solve such a question, we proposed to reconsider the subtle mechanical underpinnings of the action, so that to distinguish what is volitional and when the ensuing afferent information is generated: initiating gait means to project the CoM forward (Gélat et al., 2006). Such forward projection results from the misalignment between the center of pressure (CoP) and the vertical CoM projection onto the ground, so as to produce a disequilibrium torque that accelerates the CoM in the anterior direction (Brenière et al., 1987); therefore, such “loss of equilibrium” is the actual goal of prime mover activity. Moreover, the CoP movement is a remarkable source of afferent kinesthetic and cutaneous information (Meyer et al., 2004), thus what it may be called APA/EPA includes a time window in which this sensory information can already lead to the feedback control of the motor command. However, this contrasts with the definition of APA/EPA as feedforward programmed motor activities. Thus, the motor activities planned in a merely feedforward manner should be looked for before the first CoP displacement. So far, Sol and TA activities have been indicated as APAs because their timing excludes any contribution of feedback afferent information. However, this single criterion is not sufficient to define Sol and TA activities as APAs. In fact, in view of their role in causing the initial CoM displacement, i.e., the gait initiation goal, TA and Sol should rather be the prime movers.

At this point, it becomes crucial to redefine what should it be the role of APAs in gait initiation. Arguing that the disequilibrium torque pushing the CoM forward is mechanically equivalent to a forward force acting at the pelvis level, it follows that such concentrated force should be distributed throughout the body so that all body segments move homogeneously. This requires “fixation chains” linking the various body segments to the pelvis, which now acts as the “support point.” This leads the role of APAs in gait initiation back to the more general view, i.e., to prevent the perturbations caused by the focal movement.

According to this novel viewpoint, we would like to propose that (i) Sol and TA act as prime movers because they directly produce the intended forward displacement of the CoM, and (ii) APAs, in their literal acceptation, should be searched for before (or around) the backward CoP shift, in those muscles that link the various body segments to the pelvis. In this aim, we analyzed gait initiation in healthy subjects, searching for activities in upper and lower trunk muscles, accompanying in time the onset of the backward CoP shift.

## Materials and Methods

### Participants and Experimental Protocol

We enrolled 15 healthy subjects (6 females) with the age of 23 ± 4 years (mean ± SD), the height of 1.73 ± 0.08 m, and the weight of 68.8 ± 12.7 kg. All participants were right-footed, as ascertained by asking them which leg they used for kicking a ball, stepping up on a chair, and leading off in the long jump, as well as by observing the limb they used to start walking.

All subjects were free of any musculoskeletal or neurological dysfunction and gave their written informed consent to the study. The experimental procedure was carried out in accordance with the standards laid down in the Declaration of Helsinki and approved by the “Comitato Etico di Ateneo dell'Università degli Studi di Milano” (counsel 6/19). Subjects were asked to perform a gait initiation task: standing on a force plate for 10 s and then begin walking at their natural speed in response to a vocal prompt. After collecting a minimum of 10 trials starting with the preferred right foot, subjects were asked to start walking with the contralateral non-preferred foot, for a minimum of 10 more trials.

### Recordings

A dynamometric force plate (9286AA, KISTLER, Winterthur, Switzerland) was used to compute the CoP position. Wireless probes (FREEEMG 1000, BTS, Italy) were employed bilaterally to record the surface electromyographic (EMG) activity of TA, Sol, Rectus Abdominis (RA), Obliquus Abdominis (OA), Erector Spinae at L2 vertebra (ES-L), Erector Spinae at T3 (ES-T), Semispinalis Capitis (SC), and Deltoideus Anterior (DA). Electrodes were placed according to the Surface Electromyography for the Non-Invasive Assessment of Muscles (SENIAM) guidelines (Hermens et al., 2000). Body kinematics was recorded through an eight-camera optoelectronic system (SMART-DX, BTS, Milan, Italy) using a full-body marker set (Ferrari et al., 2008), which allowed estimating the CoM and the heel-off events. Synchronous data acquisition was accomplished by the SMART-DX workstation; the sampling rate being 100 Hz for optoelectronic cameras, 400 Hz for dynamometric signals, and 1,000 Hz for EMG.

### Data Processing

**Figure 1:**
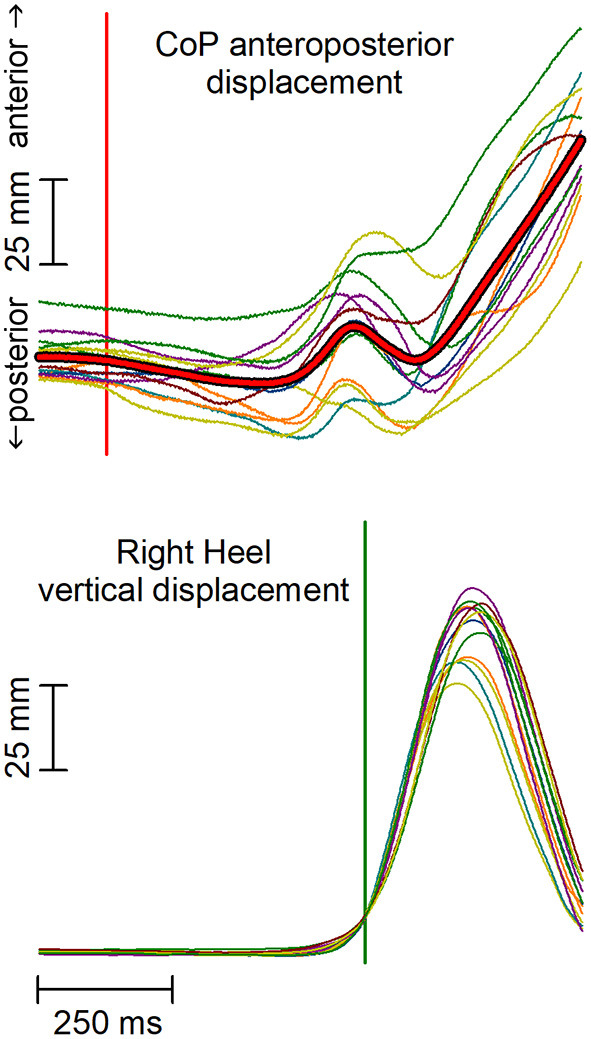
Anterior-posterior (AP) displacement of the center of pressure (CoP) and vertical displacement of the leading heel in a representative subject. Thin traces represent single trials, which are time aligned to the heel-off (vertical green line). Red thick trace in the upper plot represents the average CoP displacement. Notably, the high trial-by-trial variability of CoP makes the automatic identification of the CoP onset difficult and error-prone in each single trial. The vertical red line marks the CoP shift onset as identified on the average CoP trace.

The analysis aimed at highlighting APAs accompanying the first backward displacement of the CoP, which marks the onset of the afferent signals produced by the voluntary recruitment of the gait prime mover muscles. The data analysis approach replicated that used by Marchese et al. (2019). In brief, the raw EMG data were high-pass filtered with a zero-phase shift sixth-order elliptic filter and a cut-off frequency of 50 Hz, to remove movement artifacts. For abdominal muscles, the cut-off frequency was set to 150 Hz to remove the cardiac artifacts. Traces were then full-wave rectified, without applying any smoothing, then time-aligned to the heel-off of the leading foot, and averaged across trials. The heel-off was automatically identified as the time in which the heel marker raised 10 mm above its quiet standing value; this signal was chosen for time alignment instead of the CoP shift due to the much higher trial-by-trial variability of the CoP traces (Figure 1). The same averaging procedure was applied to CoP and CoM traces. All subsequent measurements were taken by a software algorithm on the averaged traces and visually validated.

First, the onset of the backward CoP shift was extracted by seeking for the first point in which the trace fell below −2 SD with respect to the mean level recorded from 3 to 1 s prior to heel-off and remained below that value for at least 50 ms. When this criterion was met, the algorithm searched backward the point where the trace started deviating from such mean level. Time 0 was then assigned to CoP onset.

Thereafter, the same algorithm was applied to identify the EMG changes by (i) integrating the trace with a 25-ms running average window, (ii) measuring the mean level and SD of the trace from 3 to 1 s before the CoP shift (baseline time window), and (iii) setting the searching threshold to mean +2 SD for excitation and mean −2 SD for inhibition. The algorithm search was restrained into two time windows: from −300 to +100 ms with respect to CoP onset, in order to identify APAs, and from +100 ms to heel-off, in order to identify those later changes in EMG activity of postural muscles that precede the heel-off.

### Statistical Analysis

Due to the lack of appreciable APAs and/or later EMG changes in one or more muscles of several subjects, it was not possible to fill up a complete repeated measures design on latency. Therefore, such values were not statistically compared but only reported as means and SD, together with the occurrence of such APAs. The occurrence was expressed both in terms of the number of subjects showing APAs and in percentage with the corresponding 95% CI (Tables 1–4), calculated according to the Clopper–Pearson “exact” method.

**Table 1:** Mean latency of anticipatory postural adjustments (APAs) with SD (in parentheses) for each postural muscle when starting gait with the preferred limb.

**Starting gait with the preferred right limb**				
**Muscle**	**Leading side**		**Trailing side**	
	**Mean latency (ms)**	**Occurrence**	**Mean latency (ms)**	**Occurrence**
RA	−33 (101)	*N* = 11, 73% (45–92)	−27 (48)	*N* = 11, 73% (45–92)
OA	−54 (101)	*N* = 13, 87% (59–98)	−82 (95)	*N* = 12, 80% (52–96)
ES-L	−104 (89)	*N* = 9, 60% (32–84)	−75 (96)	*N* = 10, 67% (38–88)
ES-T	−120 (75)	*N* = 7, 47% (21–73)	−32 (69)	*N* = 7, 47% (21–73)
			−113	*N* = 1, 7% (0.2–32)
SC	−38 (85)	*N* = 5, 33% (12–62)	−112 (88)	*N* = 3, 20% (4–48)
	−75	*N* = 1, 7% (0.2–32)	−10 (123)	*N* = 2, 13% (1.7–40)
DA	−10 (48)	*N* = 6, 40% (16–68)	93 (88)	*N* = 2, 13% (1.7–40)

*Latency was measured with respect to the onset of the backward center of pressure (CoP) shift, negative values indicate that the APA started before the CoP shift, and N is the number of subjects that showed the APA, also expressed as a percentage with 95% CI (in parentheses). The color shows the pattern: red for excitation and blue for inhibition*.

**Table 2:** Mean latency of APAs with SD (in parentheses) for each postural muscle when starting gait with the non-preferred limb.

**Starting gait with the non-preferred left limb**				
**Muscle**	**Leading side**		**Trailing side**	
	**Mean latency (ms)**	**Occurrence**	**Mean latency (ms)**	**Occurrence**
RA	−68 (74)	*N* = 6, 40% (16–68)	−79 (101)	*N* = 9, 60% (32–84)
OA	−51 (94)	*N* = 7, 47% (21–73)	−74 (83)	*N* = 8, 53% (27–79)
ES-L	−106 (44)	*N* = 6, 40% (16–68)	16 (70)	*N* = 5, 33% (12–62)
	24	*N* = 1, 7% (0.2–32)	−72 (39)	*N* = 2, 13% (2–40)
ES-T	−48 (82)	*N* = 6, 40% (16–68)	−17 (65)	*N* = 5, 33% (12–62)
SC	0.5 (68)	*N* = 4, 27% (8–55)	69 (35)	*N* = 2, 13% (2–40)
	83 (18)	*N* = 2, 13% (2–40)	−40	*N* = 1, 7% (0.2–32)
DA	−58 (54)	*N* = 3, 20% (4–48)	66 (13)	*N* = 2, 13% (2–40)

*Latency was measured with respect to the onset of the backward CoP shift, negative values indicate that the APA started before the CoP shift, and N is the number of subjects that showed the APA, also expressed as a percentage with 95% CI (in parentheses). The color shows the pattern: red for excitation and blue for inhibition*.

**Table 3:** Mean latency of the change in electromyographic (EMG) activity occurring between CoP onset and heel-off, with SD (in parentheses), when starting gait with the preferred limb.

**Starting gait with the preferred right limb**				
**Muscle**	**Leading side**		**Trailing side**	
	**Mean latency (ms)**	**Occurrence**	**Mean latency (ms)**	**Occurrence**
RA	404 (108)	*N* = 13, 87% (59–98)	498 (131)	*N* = 12, 80% (52–96)
OA	397 (90)	*N* = 15, 100% (78–100)	425 (158)	*N* = 15, 100% (78–100)
ES-L	308 (159)	*N* = 15, 100% (78–100)	333 (119)	*N* = 14, 67% (38–88)
ES-T	258 (132)	*N* = 13, 87% (59–98)	380 (87)	*N* = 13, 87% (59–98)
SC	329 (145)	*N* = 13, 87% (59–98)	390 (114)	*N* = 10, 67% (38–88)
			371	*N* = 1, 7% (0.2–32)
DA	340 (117)	*N* = 13, 87% (59–98)	441 (84)	*N* = 11, 73% (45–92)

*Latency was measured with respect to the onset of the backward CoP shift, all values are positive since these EMG changes started after the CoP shift, N is the number of subjects that showed the EMG change, also expressed as a percentage with 95% CI (in parentheses). The color shows the pattern: red for excitation and blue for inhibition*.

**Table 4:** Mean latency of the change in EMG activity occurring between CoP onset and heel-off, with SD (in parentheses), when starting gait with the non-preferred limb.

**Starting gait with the non-preferred left limb**				
**Muscle**	**Leading side**		**Trailing side**	
	**Mean latency (ms)**	**Occurrence**	**Mean latency (ms)**	**Occurrence**
RA	336 (106)	*N* = 12, 80% (52–96)	373 (144)	*N* = 12, 80% (52–96)
OA	375 (107)	*N* = 13, 87% (59–98)	392 (118)	*N* = 14, 67% (38–88)
ES-L	325 (98)	*N* = 12, 80% (52–96)	330 (79)	*N* = 13, 87% (59–98)
ES-T	341 (93)	*N* = 12, 80% (52–96)	384 (111)	*N* = 11, 73% (45–92)
SC	360 (143)	*N* = 12, 80% (52–96)	477 (94)	*N* = 6, 40% (16–68)
DA	386 (102)	*N* = 7, 47% (21–73)	402 (158)	*N* = 10, 67% (38–88)

*Latency was measured with respect to the onset of the backward CoP shift, all values are positive since these EMG changes started after the CoP shift, and N is the number of subjects that showed the EMG change, also expressed as a percentage with 95% CI (in parentheses). Red color indicates excitation*.

With regard to the APA occurrence data in each postural muscle and subject (i.e., 1 for presence and 0 for absence), the Aligned Rank Transformation Tool (Wobbrock et al., 2011) was applied to correct for the non-Gaussianity. The transformed data were then analyzed by a three-way ANOVA with repeated measures. The factors were *Muscle* (RA vs. OA vs. ES-L vs. ES-T vs. SC vs. DA), *Starting limb* (right vs. left) and *Body side* (leading vs. trailing), Thereafter, Tukey *post-hoc* tests were run on significant effects. The significance threshold was set at *p* < 0.05.

## Results

### EMG Activity Preceding the Backward CoP Shift

#### Starting Gait With the Preferred Limb

**Figure 2:**
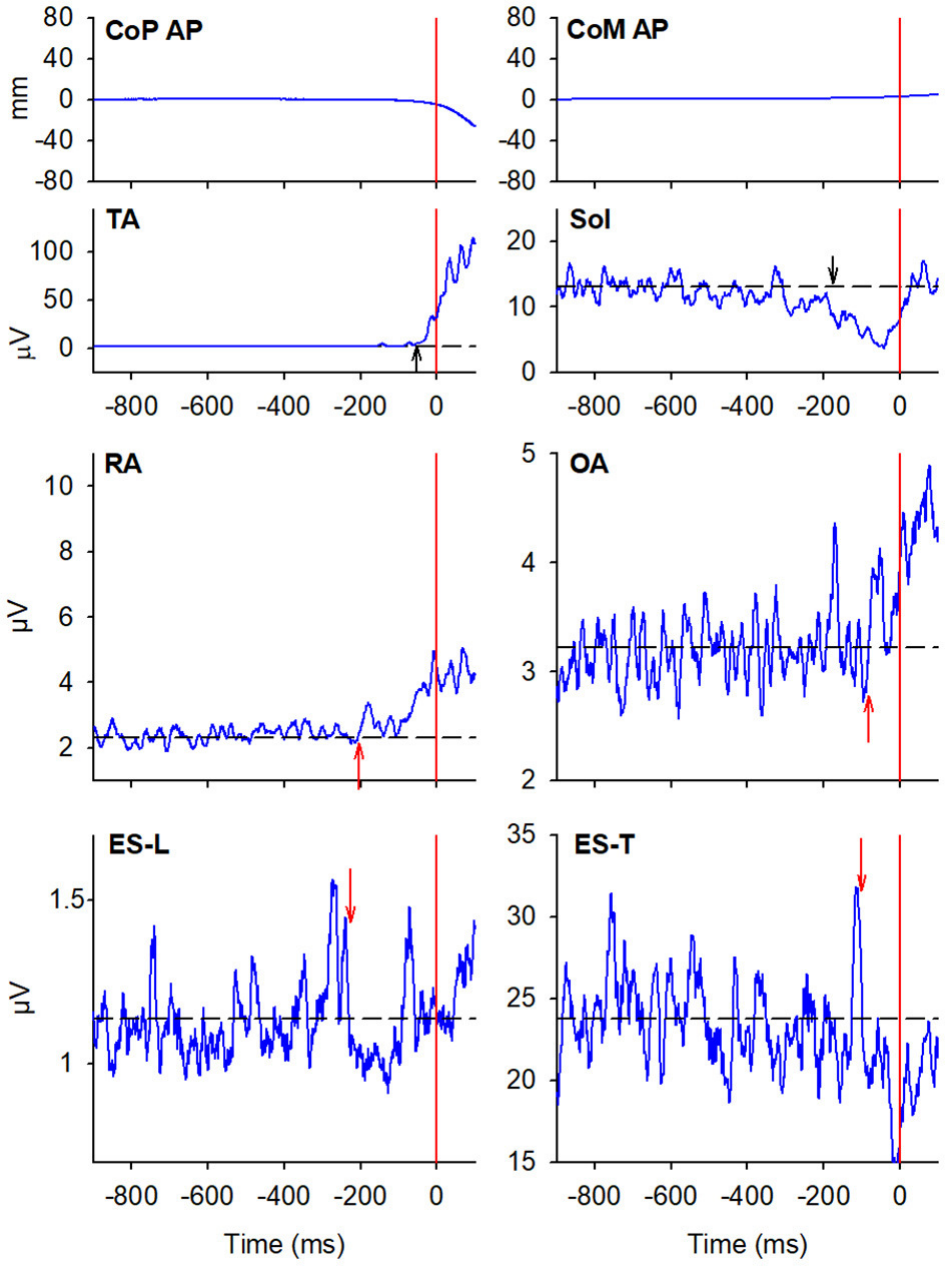
The two uppermost plots display the AP displacements of CoP and center of mass (CoM) in a representative subject when starting gait. Vertical red lines at time 0 mark the onset of the backward CoP shift. The remaining plots show the EMG activity recorded in the prime movers Tibialis Anterior (TA) and Soleus (Sol), as well as in trunk postural muscles Rectus Abdominis (RA), Obliquus Abdominis (OA), Erector Spinae at L2 vertebra (ES-L), and Erector Spinae at T3 (ES-T) of the leading side (right). Arrows in each EMG plot mark the onset of excitation (upward arrow) or inhibition (downward arrow). Black dashed lines represent the average muscle activity in the baseline time window.

**Figure 3:**
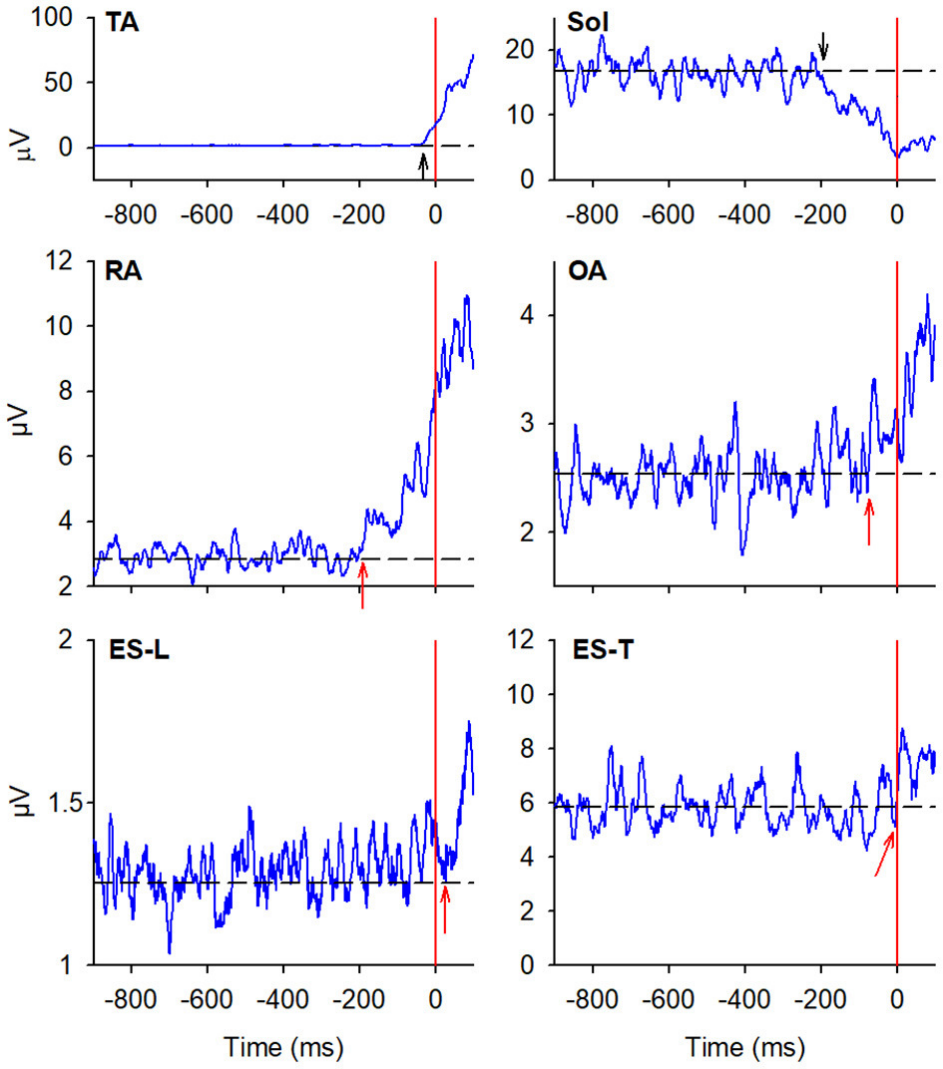
EMG activity in the prime mover (TA and Sol) and trunk postural muscles (RA, OA, ES-L, and ES-T) recorded on the trailing side (left) of the same representative subject as in Figure 2. Vertical red lines at time 0 mark the onset of the backward CoP shift. Arrows in each EMG plot mark the onset of excitation (upward arrow) or inhibition (downward arrow). Black dashed lines represent the average muscle activity in the baseline time window.

In the majority of the subjects, APAs were observed in the EMG activity of the dorsal and ventral muscles of the trunk. In particular, on the side of the leading limb (right), RA and OA were activated while ES-L and ES-T were inhibited before the backward CoP shift (Figure 2). Such shift was driven by the coordinated bilateral inhibition of Sol and activation of TA, which thus act as prime mover muscles (Figures 2, 3). On the contralateral side, both abdominal and spinal muscles mainly showed excitatory APAs accompanying the CoP shift, while TA and Sol maintained the same pattern exhibited on the leading side (Figure 3). With regard to the SC muscles, APAs could be recorded in less than half of the subjects who mainly showed an inhibitory APA on the leading side. Instead, on the trailing side, three subjects showed excitatory APAs and two displayed inhibitory ones. Even fewer subjects showed APAs in DA muscles, who were excited on both body sides. Table 1 reports the average latency of APAs for each postural muscle, the number of subjects that showed them, and the resulting percentage of occurrence with its 95% CI.

#### Starting Gait With the Non-preferred Limb

When subjects were asked to start gait with their non-preferred limb (left), overall, they showed the same APA pattern observed when starting with the right limb (Table 2). RA and OA developed excitatory APAs on both body sides while ES-L and ES-T mainly showed inhibitory APAs on the leading side (now the left one) and excitatory APAs on the trailing side. Considering SC, APAs were mainly inhibitory on the leading side and excitatory on the contralateral one, but such prevalence was less strong than when starting gait with the preferred limb (Tables 1, 2). Finally, very few subjects showed APAs in DA, excitatory in all cases. It is apparent from Tables 1 and 2 that the occurrence of APAs, regardless of their sign, decreased when passing from abdominal (RA and OA) to dorsal (ES-L and ES-T) to upper trunk muscles (SC and DA). Moreover, APA occurrence was lower when starting gait with the non-preferred limb vs. the preferred one.

Statistics confirmed these observations by revealing a main effect of *Muscle* (*F*_5, 70_ = 5.75, *p* < 0.0002) and *Starting limb* (*F*_1, 14_ = 14.07, *p* = 0.0021), while the main effect of *Body side* and all *interactions* were non-significant (in all cases, *p* > 0.48). *Post hoc* on the *Muscle* effect discovered that APA occurrence was lower in DA than in RA, OA, and ES-L (*p* < 0.013) as well as in SC than in OA (*p* = 0.025). Similar conclusions were obtained if considering only those APAs whose sign was prevalent (e.g., only the three excitatory APAs in the SC of the trailing side when starting with the right limb). In this case, the APA occurrence in SC was significantly lower than in RA, OA, and ES-L (*p* < 0.032). In conclusion, a structured pattern of APAs was observed in trunk muscles before the first backward CoP shift. However, such APAs were less frequent than the muscular actions which current literature reports to occur during the imbalance phase (i.e., between CoP shift and heel-off).

### EMG Activity Before Heel-Off

In general, EMG changes preceding the heel-off were larger than APAs preceding the CoP shift and could be easily observed in more subjects, especially in ES, SC, and DA muscles. Moreover, such changes were excitatory in all but one case.

#### Starting Gait With the Preferred Limb

**Figure 4:**
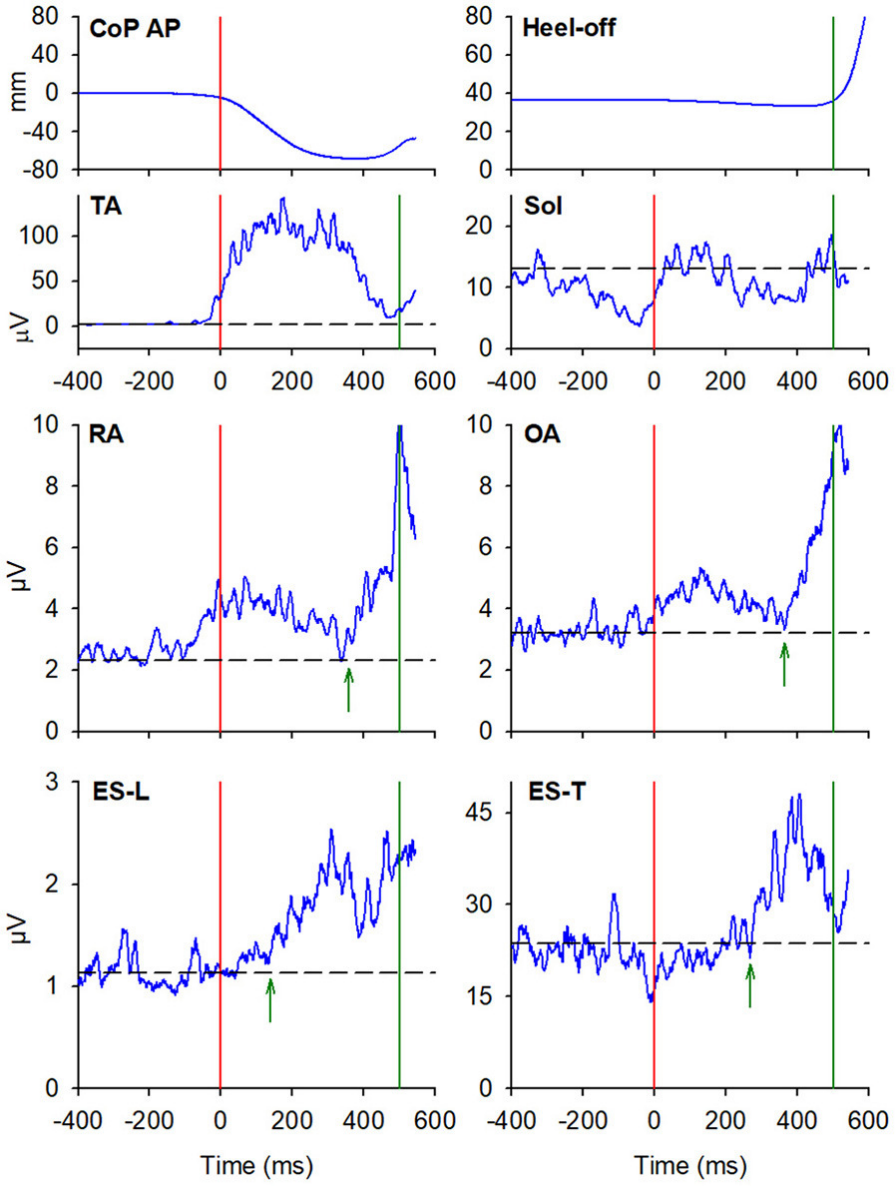
The two uppermost plots display the AP displacements of CoP and the vertical displacement of the leading heel, in the same representative subject of Figures 2, 3, when starting gait. Vertical red lines at time 0 mark the onset of the backward CoP shift while vertical green lines mark the heel-off. The remaining plots show the EMG activity recorded in the prime mover (TA, Sol) and trunk postural muscles (RA, OA, ES-L, ES-T) of the right leading side. Green arrows in each plot mark the onset of the changes in EMG activity that follow the CoP onset and precede the heel-off. Black dashed lines represent the average muscle activity in the baseline time window.

**Figure 5:**
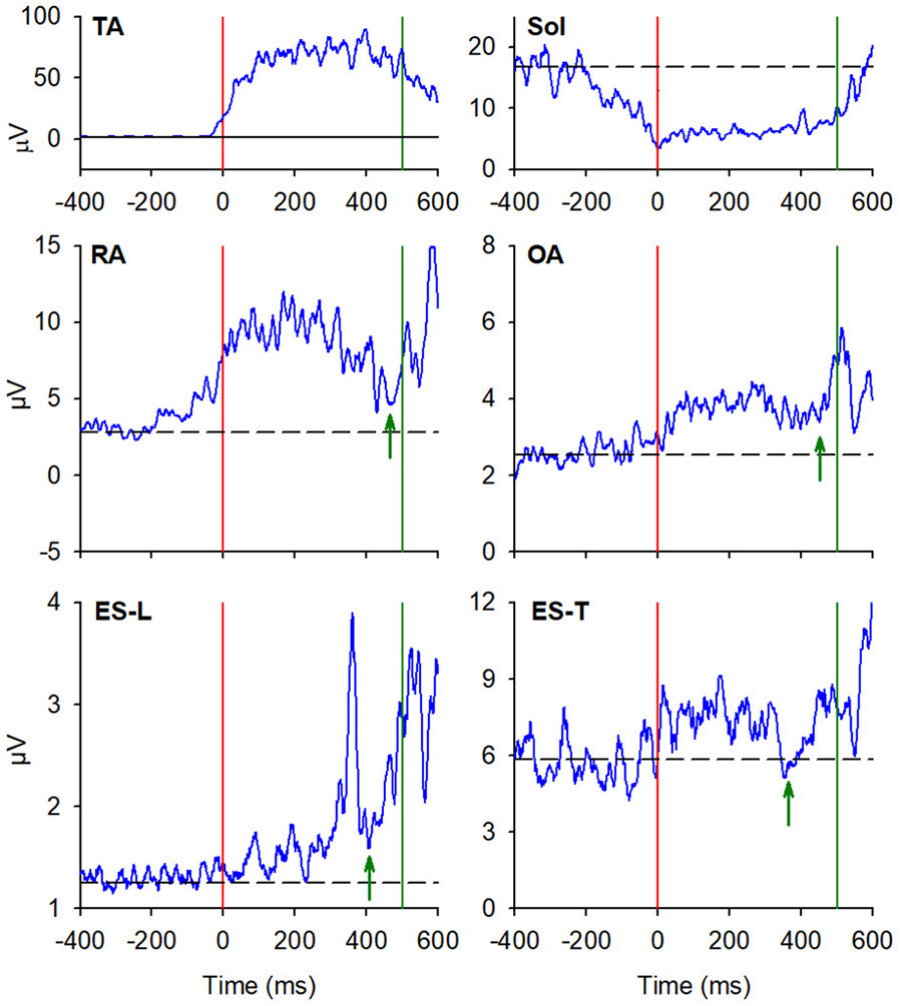
EMG activity in the prime mover (TA and Sol) and trunk postural muscles (RA, OA, ES-L, and ES-T) recorded on the trailing left side of the same representative subject of Figures 2, 3. Vertical red lines at time 0 mark the onset of the backward CoP shift while vertical green lines mark the heel-off. Green arrows in each plot mark the onset of the second changes in EMG activity that, once more, follow the CoP onset and precede the heel-off. Black dashed lines represent the average muscle activity in the baseline time window.

In almost all the experimental subjects, another change in muscular activity preceded the heel-off event. On the leading right side, in RA and OA, a second EMG burst anticipated the heel-off and followed that linked to the CoP shift (see above). Instead, the inhibitory APA observed in ES-L and ES-T, shown in Figure 2, turned into excitation before heel-off (Figure 4; Table 3). Also on the contralateral side, almost all subjects showed a new burst after the excitatory APA, in both ventral (RA and OA) and dorsal (ES-L and ES-T) muscles (Figure 5; Table 3). With regard to the SC muscles, the new change in EMG activity was mainly excitatory on both body sides and was observed in more subjects than before the CoP shift (Tables 1, 3). The increase in occurrence was also evident in DA muscles, in which the new EMG change was also excitatory.

#### Starting Gait With the Non-preferred Limb

When starting gait with the Non-preferred left limb, subjects generally showed the same pattern of excitatory EMG changes preceding heel-off as that observed when starting with the preferred limb (Table 4 vs. Table 3). However, the occurrence of such EMG changes was somewhat lower, replicating what happened for the APAs accompanying the CoP shift (Table 2 vs. Table 1).

## Discussion

To the best of our knowledge, this is the first study that systematically describes changes in trunk muscle activities preceding the backward CoP shift. Based on these observations, we reconsidered the organization of the posturo-kinetic chain related to gait initiation, in which the Sol and TA activity should no longer be considered APAs but rather as the expression of the motor command to prime movers.

A possible limitation of this study is that we did not apply EMG amplitude normalization, which prevented us from showing average traces from the entire population. Our choice was supported by the difficulty of obtaining reliable standard recordings from which to extract normalization factors. In fact, maximal voluntary contraction is particularly difficult in trunk muscles due to complex muscle synergies. Furthermore, considering that some of the recorded muscles are silent in quiet standing, using activity during that period as a normalization factor would actually be misleading.

Nevertheless, the results of this study not only confirmed the known postural activity preceding heel-off but also showed the existence of a structured pattern of anticipatory activities linked to the CoP shift, i.e., well before (about 400 ms) the muscular activity preceding heel-off. Taking into account that no gait-related afferent signals should precede the CoP shift, the first EMG changes observed in trunk muscles should be the expression of feedforward postural adjustments, i.e., APAs. Furthermore, these activities are also consistent with gait initiation mechanics. As stated in the “Introduction” section, the backward CoP shift exploits the body weight to produce a disequilibrium torque that pushes the pelvis forward and the feet backward. If this action occurred on a low friction surface, such as on ice, all the body would rotate forward around the pelvis (where CoM lies) and fall, without a net horizontal shift of the CoM. On the ground, instead, the frictional reaction force supports a forward CoM shift, promoting the first step. The force acting at the pelvis is then distributed throughout the body so that all segments move forward homogeneously. This requires APAs to build up “fixation chains” between the various body segments and the pelvis, which now acts as the “support point.” In fact, the bilateral activation of RA and OA, coupled to the reciprocal antagonist action of ES-L and ES-T on the trailing vs. leading side, stiffen the trunk to follow the forward pelvis displacement and its simultaneous rotation toward the trailing side. Thus, in view of their timings and mechanical actions, the trunk muscle activities linked to the CoP shift should be the actual APAs of gait initiation, a view that leads back such APAs to the more general definition, i.e., APAs aim at preventing the perturbations acting on the body. The same stiffening action may also be observed in the DA muscles: their activity observed in the earliest phase of gait is seemingly aimed at contrasting the arm inertia and at transmitting the forward motion of the trunk also to the upper limb. In fact, during gait initiation, the arm swing does not yet occur; it will become apparent only during walking. In this view, DA acts as a postural muscle, in the same way as the trunk muscles.

So far, only a few studies have searched for activities in trunk muscles linked to gait initiation. Despite the recordings reported in some of those studies (Assaiante et al., 2000; Wang et al., 2005, 2017; Ceccato et al., 2009; Rum et al., 2021) encompassed the initial backward shift of CoP, no description was provided for EMG activities preceding such shift, while changes occurring between CoP shift and heel-off were systematically reported. Assaiante et al. (2000) studied the development of postural chains in children, showing an excitation occurring in both extensor and flexor muscles of the trunk, only on the swing side, about 200 ms before heel-off, i.e., 300 ms after the CoP shift. Ceccato et al. (2009) later confirmed this finding in adults, by bilaterally recording ES muscles at several spinal levels and reporting that, in a comparable time window, excitatory EMG changes occurred on the swing side. Instead, Maslivec et al. (2018) reported a bilateral excitation in sternocleidomastoid and ES muscles (at T9 and L3 level), again after the backward CoP shift, with a larger delay in sternocleidomastoid in older subjects. The bilateral activation of cervical and thoracic ES in elder subjects is appreciable also in the study by Rum et al. (2021), again about 200 ms after the CoP shift. Wang et al. (2005, 2006, 2017) dedicated several studies to this topic. In some studies, they considered a time window including the first CoP shift and recorded activities in trunk muscles on the right side (Wang et al., 2005, 2006, 2017). Illustrations in these reports confirmed that dorsal and ventral muscles are excited before heel-off, both when starting with the dominant right limb and with the contralateral one.

Notably, all the above mentioned studies described muscular activities occurring before heel-off but after the CoP shift; none of them explicitly reported pre-CoP shift activities similar to those we described in this study. However, considering that the pre-heel-off activities reported in the literature are comparable to what we have shown in Figures 4, 5, we feel confident that our methodological approach and data analysis provided reliable results. Since we observed that APAs before the CoP shift are apparently smaller, less frequent, and less systematic than the excitations preceding heel-off (compare Tables 3, 4 with Tables 1, 2), maybe the above cited authors simply neglected them.

In our experiments, the occurrence of APAs in trunk muscles was significantly lower when starting gait with the non-preferred limb and decreased when passing from abdominal to dorsal and then to upper trunk muscles. In this regard, the higher APA occurrence when starting gait with the preferred foot agrees with studies showing a relationship between APA pattern and lateral dominance (Teyssèdre et al., 2000; Bruttini et al., 2016). Being the result of the feedforward control, APAs should be based on the previous motor learning processes (Massion, 1998); from this perspective, starting gait with the preferred foot is seemingly the most trained motor plan, also from a postural point of view.

Finally, a specific comment deserves TA and Sol activities, which are repeatedly reported in the literature as anticipatory adjustments preceding the CoP shift (Brenière and Do, 1991; Crenna and Frigo, 1991; refer to Yiou et al., 2017 for a review). As already stated in the “Introduction” section, initiating gait means to project the CoM forward (Gélat et al., 2006). In this regard, the bilateral inhibition of the tonic Sol activity followed by the excitation of both TAs actually drives the backward CoP shift and the ensuing CoM displacement. Therefore, TA and Sol act as prime movers, not as postural muscles. In fact, the anticipation of muscle recruitment with respect to the onset of afferent signals (i.e., CoP shift) and the fact that such recruitment scales with the intended gait velocity (Crenna and Frigo, 1991; Lepers and Brenière, 1995) are two shared properties of both APAs and prime mover activities. Thus, such properties do not allow distinguishing between the two categories. Instead, the functional differences should be searched for in their mechanical roles: counterbalancing the perturbation (APAs) vs. driving the focal movement (prime movers).

## Data Availability Statement

The raw data supporting the conclusions of this article will be made available by the authors, without undue reservation.

## Ethics Statement

The studies involving human participants were reviewed and approved by Comitato Etico di Ateneo dell'Università degli Studi di Milano (counsel 6/19). The patients/participants provided their written informed consent to participate in this study.

## Author Contributions

PC: conceptualization, funding acquisition, and supervision. VF, FB, SM, and RE: investigation and formal analysis. VF and FB: writing—original draft preparation. VF, FB, SM, RE, and PC: writing—review and editing. All authors have read and agreed to the published version of the manuscript.

## Funding

This study has been supported by internal funds from Università degli Studi di Milano and by funds from Fondazione Pierfranco e Luisa Mariani.

## Conflict of Interest

The authors declare that the research was conducted in the absence of any commercial or financial relationships that could be construed as a potential conflict of interest.

## Publisher's Note

All claims expressed in this article are solely those of the authors and do not necessarily represent those of their affiliated organizations, or those of the publisher, the editors and the reviewers. Any product that may be evaluated in this article, or claim that may be made by its manufacturer, is not guaranteed or endorsed by the publisher.
